# Peripheral inflammation is linked with emotion and mental health in people with obesity. A “head to toe” observational study

**DOI:** 10.3389/fendo.2023.1197648

**Published:** 2023-07-14

**Authors:** Charalampia Amerikanou, Evdokia Valsamidou, Stamatia-Angeliki Kleftaki, Aristea Gioxari, Konstantinos Koutoulogenis, Maria Aroutiounova, Ioannis Stergiou, Andriana C. Kaliora

**Affiliations:** ^1^Department of Nutrition and Dietetics, School of Health Science and Education, Harokopio University, Athens, Greece; ^2^Department of Nutritional Science and Dietetics, School of Health Science, University of the Peloponnese, Kalamata, Greece; ^3^Diabetes Outpatient Department, General Hospital G. Gennimatas, Thessaloniki, Greece

**Keywords:** obesity, metabolic syndrome, inflammation, oxidative stress, mental health

## Abstract

**Background:**

Obesity is a significant worldwide health problem that is linked with mental health. The elucidation of the possible overlapping biochemical mechanism(s) involved in inflammation and oxidative stress is imperative to better understand and address obesity and related metabolic disorders. The aim of the study was to investigate the associations between inflammatory and oxidative stress profiles with parameters that reflect metabolic, emotional, and mental health in a Greek metabolically unhealthy obese cohort.

**Methods:**

In total, 122 metabolically unhealthy people with obesity were recruited. Anthropometric measurements, biochemical, inflammatory and oxidative stress markers were assessed. Quality of life was evaluated through questionnaires for insomnia, self-esteem, depression, physical and mental health.

**Results:**

The inflammatory biomarker tumor necrosis factor-alpha (TNF-α) and the ratio oxidized low-density lipoprotein/low-density lipoprotein (oxLDL/LDL) were higher in hypertensive (p=0.002, p=0.001 respectively) and hyperglycemic subjects (p=0.017, p=0.001 respectively). Furthermore TNF-α (p<0.001), oxLDL/LDL (p<0.001) and oxLDL/high-density lipoprotein (HDL) (p=0.016) increased significantly with the increase of metabolic syndrome components. Finally, a negative association between interleukin-6 (IL-6) and Rosenberg Self-Esteem Scale (Beta=-0.019, p=0.019) and a positive association between TNF-α and the Center for Epidemiologic Studies Depression Scale Revised (Beta=0.003, p=0.015) were found.

**Conclusions:**

The results of the study suggest that obesity-related systemic inflammation is associated with worse self-esteem and depression symptoms, indicating an overlapping mechanism which can be utilized to the management of obesity.

## 1 Introduction

Overweight and obesity have increased dramatically during the last years, reaching pandemic levels and constituting a major public health issue associated with non-communicable diseases, such as type 2 diabetes mellitus (T2DM), cardiovascular diseases (CVDs), non-alcoholic fatty liver disease (NAFLD) and several types of cancers (1). The Global Burden of Disease 2015 study showed that 603.7 million adults were living with obesity worldwide and high body mass index (BMI) contributed to 4.0 million deaths and 120 million disability-adjusted life-years in 2015 (2). A high increase in the prevalence of obesity from 1980 to 2019 was observed with a raise from 4.6% to 14.0% globally and from 8.4% to 20.0% in Europe (3). In Greece, according to the World Health Organization, the 2016 age-standardized prevalence of overweight and obesity among adults was 62.3% and 24.9% for both sexes respectively (4).

Although the traditional definition of obesity includes the presence of excessive body fat accumulation, obesity is considered a complex and multifactorial disease, caused by several genetic, epigenetic, lifestyle and environmental factors. Inflammation and oxidative stress (OS) play a critical role and are closely connected to obesity and related metabolic abnormalities. Adipose tissue is characterized by a chronic low-grade inflammation due to the activation of the innate immune system that promotes pro-inflammatory status and OS (5). Low-grade chronic inflammation, caused by the excess presence of nutrients in circulation and tissues, known as metaflammation, is associated with monocyte recruitment, macrophage infiltration and activation of pro-inflammatory cytokines (6). Also, obesity is related to a pro-oxidative status with reactive oxygen species (ROS) generation, increased production of NADPH oxidase (NOX), decreased expression of antioxidant enzymes and dysregulated production of adipokines, such as adiponectin, and leptin (7).

Such an immune response is critical for the interaction between metabolism, inflammation and the development of obesity-associated comorbidities (6). The activation of the innate immune system in obesity promotes the production of pro-inflammatory cytokines such as leptin, tumor necrosis factor-alpha (TNF-α) and interleukin-6 (IL-6) and acute-phase proteins such as C-reactive protein (CRP) and inhibits the production of anti-inflammatory cytokines, such as adiponectin (8). Adiponectin is important for insulin sensitivity; therefore its decrease induces insulin resistance and results in T2DM. Furthermore, inflammation induces dyslipidemia, atherosclerosis and hypertension through endothelial dysfunction and increased arterial stiffness. Hypertension is further aggravated by obesity due to the impairment of pressure natriuresis (9). These pathologies are predominantly present in individuals with central obesity. Fat accumulation in the abdomen is the most prevalent component of the metabolic syndrome (MS). MS is a cluster of pathologies that includes abdominal obesity, dyslipidemia, hyperglycemia and hypertension. The aforementioned complex interplay highlights the importance of circulating biomarkers for the understanding of obesity and related metabolic abnormalities, as well as for the monitoring of disease progression.

Obesity has not only physiological implications but is also associated with mental and emotional disorders, such as depression, low self-esteem and eating disorders, leading consequently to a decreased quality of life (10). The link between obesity and mental health is bidirectional and when they happen concurrently the one exacerbates the other. Psychological interventions, including behavioral therapy, have exhibited promising results in the management of obesity (11). Furthermore, there is growing evidence that inflammation is associated with mental health (12). Corroborating to this, a recent review addressed the idea that excessive body weight leads to neuroinflammation and possible cognitive dysfunction (13). Although, dietary interventions for body weight management have been shown to improve the symptoms of depression, the role of inflammation was not thoroughly examined (14).

Clearly, there is a link between obesity, inflammation and mental well-being that begs for more research. The association between inflammation and quality of life, including mental and emotional health, in metabolically unhealthy obese people has been studied previously in a Korean population, but included only a single inflammatory biomarker, CRP (15). As obesity-related-inflammation and OS produce a plethora of dysregulated markers, we hypothesized that in a cohort of metabolically unhealthy people with obesity, other circulating biomarkers may be associated with lifestyle and quality of life parameters. Therefore, the aim of this study was to explore the associations between inflammatory and OS markers, with anthropometric, biochemical and mental health parameters in a Greek metabolically unhealthy population with obesity.

## 2 Materials and methods

### 2.1 Study design

This is an observational study built on the baseline data from an intervention registered with Clinicaltrials.gov with the ID number NCT04785573 and conducted in metabolically unhealthy Greek patients with obesity. The study was approved by the Harokopio University Ethics Committee (ID protocol: 1799/13-06-2019), and was performed in accordance with the principles of the Helsinki Declaration and the Data Protection Act 1998. All subjects gave their informed consent to participate in the study which took place in Athens, Greece, between 2021 and 2022.

### 2.2 Participants

Adult males and females were included, with abdominal obesity (waist circumference (WC)>94cm for males and >80cm for females). The presence of at least one metabolic abnormality was an inclusion criterion; triglyceride (TG) level ≥150 mg/dL, high-density lipoprotein (HDL) ≤ 40 mg/dL in men or ≤50 mg/dL in women, increased blood pressure ≥130/85 mm Hg, elevated fasting blood sugar ≥100 mg/dL. The main exclusion criteria were pregnancy, lactation, and untreated thyroid disease, use of supplements within 3 months before recruitment, drug and/or alcohol abuse, psychiatric and mental disorders. Also, subjects should have a stable body weight for at least 3 months prior randomization to the study and a moderately active lifestyle.

### 2.3 Clinical assessments and anthropometric measurements

Detailed medical history was obtained including personal, family, medical history, and medication. Body weight was measured to the nearest kilogram with a flat scale, height to the nearest centimeter with a stadiometer (Seca Mode 220, Hamburg, Germany) and BMI was calculated using weight in kg divided by the square of height in meters. WC was measured with a non-stretch but flexible tape on minimal clothing. Body composition was analyzed with bioelectrical impedance analysis (Tanita BC-418, Tokyo, Japan) and included body fat, fat-free mass (FFM), total body water (TBW) and visceral fat level (VFL).

### 2.4 Biochemical measurements

Twenty mL of blood was collected after an overnight fast. After centrifugation (3000 rpm, 10 min, 20◦ C) serum was isolated and was used for biochemical indices and biomarkers quantification.

Glucose, insulin, total cholesterol (TC), HDL, low-density lipoprotein (LDL), TG, serum glutamic-oxaloacetic transaminase (SGOT), serum glutamic-pyruvic transaminase (SGPT), γ-glutamyl transferase (γ-GT) and alkaline phosphatase (ALP) were measured with an automatic biochemical analyzer (Cobas 8000 analyzer, Roche Diagnostics GmbH, Mannheim, Germany).

### 2.5 Inflammatory and OS biomarkers assessment

IL-6, TNF-α, adiponectin, leptin (R&D Systems, Inc., Minneapolis, MN, USA), oxidized LDL (oxLDL) (Mercodia, AB, Uppsala, Sweden) and myeloperoxidase (MPO) (Thermo Fisher Scientific Inc., Waltham, MA, USA) were assessed in serum samples (n=122) applying sandwich enzyme-linked immunosorbent assay (ELISA). All measurements were performed in duplicates. CRP was measured with an automatic biochemical analyzer (Cobas 8000 analyzer, Roche Diagnostics GmbH, Mannheim, Germany). In order to compute oxidized to non-oxidized lipoprotein ratios, mg/dL of LDL and HDL were converted to mmol/L and oxLDL/LDL and oxLDL/HDL were calculated.

### 2.6 Quality of life assessment

Quality of life was assessed using validated questionnaires. Athens Insomnia Scale (AIS), a self-administered psychometric instrument was used for the evaluation of sleep quality. It consists of eight items (0-3 rate each) that evaluate sleep induction, awakenings, sleep duration and quality, well-being, functioning capacity and sleepiness during the day (16). The AIS score ranges from 0-28 with higher scores indicating worse symptoms of insomnia. The Center for Epidemiologic Studies Depression Scale Revised (CESD-R) is a widely used self-report measure for depression screening. It consists of 20 items (scoring range 0-3 and a final score from 0 to 60, scores equal to or above 16 represent a risk for clinical depression) regarding mood, somatic complaints, interactions with others, and motor functioning (17). Self-esteem was assessed by applying the 10-item Revised Rosenberg Self-Esteem scale (RSES), a highly reliable measure of global self-esteem, with a 4-point scale format ranging from strongly agree to strongly disagree and a final score from 0 to 40. Scores below 15 indicate a low self-esteem (18). Finally, the self-reported Short Form-12 (SF-12) questionnaire with a physical and mental health summary scale evaluated the impact of health on participants’ everyday life with its two summary scores reporting on a mental (MCS-12) and a physical component score (PCS-12) (19).

Physical activity was assessed *via* the International Physical Activity Questionnaire Short Form (IPAQ-SF) and it is presented as the metabolic equivalent task minutes per week (MET-min/week) (20). Adherence to the Mediterranean diet was evaluated by experienced dietitians using the Mediterranean Diet score (MedDiet score) (21). Finally smoking habits were recorded.

### 2.7 Statistical analysis

The normality of variables was checked with Kolmogorov–Smirnov test. Qualitative variables are presented with absolute and relative frequencies. Continuous parametric variables are presented with mean and standard deviation (SD), whereas non-parametric ones with median and interquartile range (IQR). Comparisons of means across groups were performed with the Mann–Whitney U test or Kruskal-Wallis test. The x^2^ test was applied for categorical variables. Spearman’s correlation was used to explore the relationship between inflammatory and OS markers, and other disease parameters. For associations that were found significant, adjustments were made *via* linear regression with the application of three models. The first one was the unadjusted one, the second was adjusted for age, gender and WC and the third one was for age, gender, WC, physical activity level, smoking and the presence of any of the other MS components (hypertension, hyperglycemia, hyperlipidemia). Regression analyses were conducted after having logarithmically transformed the dependent variables. Statistical significance was set at p-value <0.05 and SPSS (version 21.0) was used for all analyses.

## 3 Results

One hundred and twenty-two Greek metabolically unhealthy adults with central obesity were included in this study. The general characteristics of the sample, including descriptive, anthropometric, clinical, lifestyle characteristics and blood markers levels are presented in **Table 1**.

**Table 1:** Characteristics of the study population.

	N=122
Age (years), mean ± SD	57.7 ± 8.8
Sex (male/female), N (%)	50 (41.0)/72 (59.0)
Anthropometric indices	
BMI (kg/m^2^), median (IQR)	34.3 (8.1)
WC (cm), mean ± SD	110.9 ± 14.1
Body fat (%), mean ± SD	40.5 ± 7.7
FFM (kg), median (IQR)	50.6 (21.0)
TBW (kg), median (IQR)	38.1 (15.0)
VFL, median (IQR)	13.0 (5.8)
Family status	
Married, N (%)	91 (74.6)
Divorced, N (%)	5 (4.1)
Single, N (%)	16 (13.1)
In a relationship, N (%)	5 (4.1)
Widowed, N (%)	5 (4.1)
MS components	
Hypertension (No/Yes/N/A), N (%)	35 (28.7)/83 (68.0)/4 (3.3)
Hyperglycemia (No/Yes/N/A), N (%)	79 (64.8)/42 (34.4)/1 (0.8)
Hyperlipidemia (No/Yes), N (%)	14 (11.5)/108 (88.5)
Lifestyle and quality of life characteristics	
Smoking, N (%)	95 (77.9)/27 (22.1)
PAL (total MET- min/week), median (IQR)	610.0 (1848.0)
AIS, mean ± SD	5.4 ± 3.9
CESD-R, mean ± SD	17.5 ± 10.1
RSES mean ± SD	30.7 ± 4.7
PCS-12, mean ± SD	45.2 ± 9.6
MCS-12, mean ± SD	49.1 ± 8.4
Meddiet score, median (IQR)	32.5 (7.0)
Biochemical markers	
Glucose (mg/dl), mean ± SD	94.6 ± 11.3
Insulin (μIU/mL), median (IQR)	13.3 (6.9)
ΤC (mg/dl), median (IQR)	204.0 (58.0)
TG (mg/dl), median (IQR)	129.5 (47.0)
HDL (mg/dl), median (IQR)	48.0 (10.8)
LDL (mg/dl), mean ± SD	128.0 ± 47.8
SGOT (iu/l), mean ± SD	17.7 ± 3.7
SGPT (iu/l), median (IQR)	18.5 (8.3)
γ-GT (iu/l), mean ± SD	19.3 ± 8.1
ALP (U/L), mean ± SD	68.5 ± 18.5
Inflammatory and OS biomarkers	
IL-6 (pg/mL), median (IQR)	2.8 (1.2)
TNF-α (pg/mL), median (IQR)	1.2 (0.3)
Leptin (ng/mL), median (IQR)	37.1 (38.9)
Adiponectin (μg/mL), median (IQR)	4.3 (8.9)
oxLDL (U/l), median (IQR)	82.3 (27.4)
oxLDL/LDL (U/mmoL), median (IQR)	25.8 (10.3)
oxLDL/HDL (U/mmoL), median (IQR)	62.7 (38.5)
MPO (ng/mL), median (IQR)	75.7 (187.5)
CRP (mg/L), median (IQR)	2.3 (4.0)

Data are expressed as counts (%) or mean ± standard deviation (SD) for parametric variables and median (interquartile range, IQR) for non-parametric variables.

BMI, body mass index; WC, waist circumference; FFM, free fat mass; TBW, total body water; VFL, visceral fat level; MS, metabolic syndrome; PAL, physical activity level as assessed by International Physical Activity Questionnaire Short Form; MET, metabolic equivalent task;AIS, Athens Insomnia Scale; CESD-R, Center for Epidemiologic Studies Depression Scale Revised; PCS-12, Physical Component Score; MCS-12, Mental Composite Score; RSES, Rosenberg Self-Esteem scale; TC, total cholesterol; TG, triglycerides; HDL, high density lipoprotein; LDL, low density lipoprotein; SGOT, serum glutamic-oxaloacetic transaminase; SGPT, serum glutamic-pyruvic transaminase; γ-GT, γ-glutamyl transferase; ALP, alkaline phosphatase; OS, oxidative stress; IL-6, interleukin-6; TNF-α, tumor necrosis factor-α; oxLDL, oxidized low density lipoprotein; MPO, myeloperoxidase; CRP, C-reactive protein.

The levels of inflammatory and OS markers between subjects with or without a metabolic component of MS (hypertension, hyperglycemia and hyperlipidemia), except from central obesity which was present in all participants, were compared (**Table 2**). Leptin, adiponectin, oxLDL, MPO and CRP levels did not differ between the different categories. TNF-α was higher in patients with hypertension (p=0.002) and hyperglycemia (p=0.017) compared to those without. Similarly, oxLDL/LDL was higher in patients with hypertension and hyperglycemia (p=0.001). Also, the comparison of IL-6 between subjects with and without hyperglycemia showed a considerable trend towards significance (p=0.069) with hyperglycemic patients exhibiting higher levels of the inflammatory cytokine.

**Table 2:** Levels of inflammatory and OS biomarkers according to the presence of MS components.

Hypertension	No (N=35)	Yes (N=83)	*p*
IL-6 (pg/mL), median (IQR)	2.1 (2.6)	2.5 (1.8)	0.278
TNF-α (pg/mL), median (IQR)	1.0 (0.4)	1.1 (0.4)	**0.002**
Leptin (ng/mL), median (IQR)	37.4 (32.1)	34.8 (39.2)	0.859
Adiponectin (μg/mL), median (IQR)	5.6 (6.7)	5.9 (9.6)	0.950
oxLDL (U/l), median (IQR)	79.2 (28.8)	70.0 (43.5)	0.177
oxLDL/LDL (U/mmoL), median (IQR)	22.0 (9.8)	27.1 (11.5)	**0.001**
oxLDL/HDL (U/mmoL), median (IQR)	54.7 (44.7)	61.7 (35.4)	0.165
MPO (ng/mL), median (IQR)	91.1 (155.0)	72.2 (114.2)	0.870
CRP (mg/L), median (IQR)	2.3 (4.7)	2.3 (4.0)	0.711
Hyperglycemia	No (N=79)	Yes (N=42)	*p*
IL-6 (pg/mL), median (IQR)	2.2 (1.6)	3.1 (3.0)	0.069
TNF-α (pg/mL), median (IQR)	1.0 (0.3)	1.2 (0.5)	**0.017**
Leptin (ng/mL), median (IQR)	36.9 (34.2)	33.1 (45.3)	0.408
Adiponectin (μg/mL), median (IQR)	6.3 (9.2)	4.1 (5.7)	0.101
oxLDL (U/l), median (IQR)	77.4 (29.1)	82.1 (38.9)	0.264
oxLDL/LDL (U/mmoL), median (IQR)	24.2 (8.8)	30.0 (12.6)	**0.001**
oxLDL/HDL (U/mmoL), median (IQR)	57.9 (30.4)	67.4 (43.1)	0.091
MPO (ng/mL), median (IQR)	85.9 (133.7)	77.9 (145.2)	0.455
CRP (mg/L), median (IQR)	2.4 (3.6)	1.9 (5.7)	0.752
Hyperlipidemia	No (N=14)	Yes (N=108)	*p*
IL-6 (pg/mL), median (IQR)	1.7 (4.2)	2.4 (1.6)	0.615
TNF-α (pg/mL), median (IQR)	1.0 (0.3)	1.1 (0.4)	0.158
Leptin (ng/mL), median (IQR)	39.4 (74.5)	34.8 (34.8)	0.163
Adiponectin (μg/mL), median (IQR)	5.1 (7.0)	6.2 (8.2)	0.527
oxLDL (U/l), median (IQR)	74.2 (41.4)	77.9 (32.9)	0.629
oxLDL/LDL (U/mmoL), median (IQR)	26.9 (8.1)	25.6 (10.4)	0.401
oxLDL/HDL (U/mmoL), median (IQR)	54.9 (24.5)	62.9 (39.8)	0.271
MPO (ng/mL), median (IQR)	109.8 (137.9)	79.2 (146.8)	0.724
CRP (mg/L), median (IQR)	4.3 (6.0)	2.3 (3.6)	0.317

Data are expressed as median (interquartile range, IQR). MS, metabolic syndrome; IL-6, interleukin-6; TNF-α, tumor necrosis factor-α; oxLDL, oxidized low-density lipoprotein; HDL, high-density lipoprotein; LDL, low-density lipoprotein; MPO, myeloperoxidase; CRP, C-reactive protein. Comparisons between groups were performed by the Mann–Whitney U test. Level of significance was set as 0.05. Significant p are in bold.

A similar pattern (**Table 3**) was detected when the levels of the above markers were compared with the number of metabolic components of MS that were present in the study population (central obesity plus; hypertension (2), hyperglycemia (3), hyperlipidemia (4)). More specifically, subjects with three metabolic components had higher levels of TNF-α and oxLDL/LDL than those with two components and subjects with four metabolic components had higher levels of the same biomarkers than those with two or three components (p< 0.001). A statistically significant difference was also found in oxLDL/LDL levels (p=0.016) with subjects with four metabolic components exhibiting higher levels than the ones with two. A trend towards significance was once again evident when IL-6 levels were compared to the presence of different numbers of MS components (p=0.052).

**Table 3:** Levels of inflammatory/OS markers according to the number of MS components.

Number of MS components	2 (N=30)	3 (N=60)	4 (N=27)	*p*
IL-6 (pg/mL), median (IQR)	1.9 (1.5)	2.5 (1.8)	3.0 (2.0)	0.052
TNF-α (pg/mL), median (IQR)	0.9 (0.3)^a,b^	1.1 (0.4)^a,c^	1.3 (0.5)^b,c^	**< 0.001**
Leptin (ng/mL), median (IQR)	37.4 (27.4)	39.1 (45.5)	21.3 (26.7)	0.272
Adiponectin (μg/mL), median (IQR)	6.0 (7.9)	6.3 (11.7)	3.9 (5.3)	0.140
oxLDL (U/l), median (IQR)	71.6 (40.0)	77.9 (27.4)	82.3 (36.8)	0.263
oxLDL/LDL (U/mmoL), median (IQR)	22.2 (9.7)^a,b^	26.0 (10.2)^a,c^	30.5 (11.2)^b,c^	**< 0.001**
oxLDL/HDL (U/mmoL), median (IQR)	53.2 (42.0)^a^	58.5 (28.0)	83.1 (42.5)^a^	**0.016**
MPO (ng/mL), median (IQR)	96.1 (162.5)	76.6 (112.8)	72.2 (151.6)	0.872
CRP (mg/L), median (IQR)	2.8 (4.2)	2.4 (3.9)	1.6 (4.3)	0.596

Data are expressed as median (interquartile range, IQR). MS, metabolic syndrome; IL-6, interleukin-6; TNF-α, tumor necrosis factor-α; oxLDL, oxidized low density lipoprotein; HDL, high-density lipoprotein; LDL, low-density lipoprotein; MPO, myeloperoxidase; CRP, C-reactive protein. Comparisons between groups were performed by the Kruskal-Wallis test. Level of significance was set as 0.05. Significant p are in bold. Different superscript levels (a, b, c) represent significant differences between groups.

The results of the correlation analysis between inflammatory and OS biomarkers and anthropometric indices, biochemical markers and quality of life questionnaires are presented in **Table 4**. Only correlations with a p<0.05 are presented. IL-6 was positively correlated with most of the anthropometric parameters (BMI: Rho=0.427, p=0.001, WC: Rho=0.424, p=0.003, % body fat: Rho=0.527, p<0.001 and VFL: Rho=0.526, p<0.001) and AIS (Rho=0.354, p=0.027). On the contrary, it was negatively correlated with HDL (Rho=-0.309, p=0.035), RSES (Rho=-0.545, p=0.002) and PCS-12 (Rho=-0.517, p=0.002). TNF-α exhibited a positive correlation with glucose (Rho=0.463, p=0.002), AIS (Rho=0.367, p=0.025) and CESD-R (Rho=0.364, p=0.041) and a negative one with RSES (Rho=-0.441, p=0.017). Leptin correlated positively with BMI (RSES (Rho=0.305, p=0.032) and % body fat (Rho=0.503, p<0.001). Adiponectin inversely correlated with SGOT (Rho=-0.339, p=0.026), SGPT (Rho=-0.518, p<0.001), and γ-GT (Rho=-0.444, p=0.003), whereas a positive correlation with PCS-12 (Rho=0.497, p=0.004) was found. OxLDL had a positive correlation with TC (Rho=0.534, p<0.001), LDL (Rho=0.493, p<0.001 and CESD-R (Rho=0.391, p=0.022). OxLDL/LDL was positively correlated with BMI (Rho=0.200, p=0.033), WC (Rho=0.239, p=0.010), TBW (Rho=0.195, p=0.044), VFL (Rho=0.391, p<0.001), glucose (Rho=0.280, p=0.003), insulin (Rho=0.257, p=0.008) and negatively with TC (Rho=-0.301, p=0.001), HDL (Rho=-0.191, p=0.041), and LDL (Rho=-0.341, p<0.001). OxLDL/HDL correlated positively with FFM (Rho=0.247, p=0.010), TBW (Rho=0.296, p=0.002), VFL (Rho=0.257, p=0.009), insulin (Rho=0.254, p=0.009), TC (Rho=0.322, p<0.001), TG (Rho=0.483, p<0.001), LDL (Rho=0.351, p<0.001), and negatively with HDL (Rho=-0.557, p<0.001). Finally, CRP positively correlated with BMI (Rho=0.464, p<0.001), WC (Rho=0.234, p<0.001), % body fat (Rho=0.429, p<0.001) and ALP (Rho=0.261, p=0.006). No significant correlations were observed between MPO and the examined parameters. 

**Table 4:** Significant correlations between inflammatory and OS biomarkers, and anthropometric indices, biochemical markers and quality of life questionnaires.

	IL-6 (pg/mL)		TNF-α (pg/mL)		Leptin (ng/mL)		Adiponectin (μg/mL)		oxLDL (U/l)		OxLDL/LDL (U/mmoL)		OxLDL/HDL (U/mmoL)		CRP (mg/L)	
	Rho	*P*	Rho	*P*	Rho	*P*	Rho	*P*	Rho	*P*	Rho	*P*	Rho	*P*	Rho	*P*
Anthropometric indices																
BMI (kg/m^2^)	0.427	0.001			0.305	**0.032**					0.200	0.033			0.464	**<0.001**
WC (cm)	0.424	0.003									0.239	0.010			0.234	**<0.001**
Body fat (%)	0.527	<0.001			0.503	**<0.001**									0.429	**<0.001**
FFM (kg)													0.247	0.010		
TBW (kg)											0.195	0.044	0.296	0.002		
VFL	0.526	<0.001									0.391	<0.001	0.257	0.009		
Biochemical markers																
Glucose (mg/dl)			0.463	**0.002**							0.280	0.003				
Insulin (μIU/mL)											0.257	0.008	0.254	**0.009**		
ΤC (mg/dl)									0.534	<0.001	-0.301	0.001	0.322	**<0.001**		
TG (mg/dl)													0.483	**<0.001**		
HDL (mg/dl)	-0.309	**0.035**									-0.191	0.041	-0.557	**<0.001**		
LDL (mg/dl)									0.493	<0.001	-0.341	<0.001	0.351	**<0.001**		
SGOT (iu/l)							-0.339	**0.026**								
SGPT (iu/l)							-0.518	**<0.001**								
γ-GT (iu/l)							-0.444	**0.003**								
ALP (U/L)															0.261	**0.006**
Quality of life questionnaires																
AIS	0.354	**0.027**	0.367	**0.025**												
CESD-R			0.364	**0.041**					0.391	**0.022**						
RSES	-0.545	**0.002**	-0.441	**0.017**												
PCS-12	-0.517	**0.002**					0.497	**0.004**								

IL-6, interleukin-6; TNF-α, tumor necrosis factor-α; oxLDL, oxidized low density lipoprotein; CRP, C-reactive protein; BMI, body mass index; WC, waist circumference; FFM, free fat mass; VFL, visceral fat level; TC, total cholesterol; TG, triglycerides; HDL, high density lipoprotein; LDL, low density lipoprotein; SGOT, serum glutamic-oxaloacetic transaminase; SGPT, serum glutamic-pyruvic transaminase; γ-GT, γ-glutamyl transferase; ALP, alkaline phosphatase; AIS, Athens Insomnia Scale; CESD-R, Center for Epidemiologic Studies Depression Scale Revised; RSES, Rosenberg Self-Esteem scale; PCS-12, Physical Component Score. Correlation analysis was performed with Spearman’s rank correlation. Level of significance was set as 0.05. Significant p are in bold.

Finally, linear regression models were applied to examine the associations of the study variables that were significant in the correlation analysis. The first applied model was an unadjusted one, the second was adjusted for age, gender and WC and the third one for age, gender, WC, physical activity level, smoking and the presence of any of the metabolic abnormalities (hypertension, hyperglycemia, hyperlipidemia). **Table 5** presents only the associations that showed a significant value in the third model. Analysis showed that IL-6 had statistically significant positive association with BMI (Beta3 ± SD= 0.012 ± 0.004, p=0.005), VFL (Beta3 ± SD= 0.023 ± 0.007, p=0.002) and a negative one with RSES (Beta3 ± SD= -0.019 ± 0.008, p=0.019). TNF-α was positively associated with CESD-R (Beta3 ± SD= 0.003 ± 0.001, p=0.015) and leptin with BMI (Beta3 ± SD= 0.013 ± 0.005, p=0.008) and % body fat (Beta3 ± SD= 0.025 ± 0.006, p<0.001). Adiponectin was inversely associated with SGPT (Beta3 ± SD= -0.010 ± 0.004, p=0.013), whereas oxLDL was positively associated with TC (Beta3 ± SD= 0.003 ± 0.000, p<0.001) and LDL (Beta3 ± SD= 0.003 ± 0.000, p<0.001). The ratio oxLDL/HDL associated positively with insulin (Beta3 ± SD= 0.006 ± 0.003, p=0.040), TC (Beta3 ± SD= 0.002 ± 0.001, p<0.001), TG (Beta3 ± SD= 0.001 ± 0.000, p<0.001), LDL (Beta3 ± SD= 0.104 ± 0.021, p<0.001) and negatively with HDL (Beta3 ± SD= -0.358 ± 0.075, p<0.001). Finally, CRP was positively associated with BMI (Beta3 ± SD= 0.025 ± 0.006, p=0.008), WC (Beta3 ± SD= 0.011 ± 0.003, p<0.001), % body fat (Beta3 ± SD= 0.021 ± 0.008, p<0.001) and ALP (Beta3 ± SD= 0.005 ± 0.002, p=0.036).

**Table 5:** Associations between inflammatory and OS biomarkers and metabolic conditions related parameters.

	Model 1^1^		Model 2^2^		Model 3^3^	
	Beta ± SE	*P*	Beta ± SE	*P*	Beta ± SE	*P*
Log IL-6						
BMI (kg/m^2^)^4^	0.016 ± 0.005	**0.001**	0.015 ± 0.005	**0.002**	0.012 ± 0.004	**0.005**
VFL^4^	0.015 ± 0.007	**0.029**	0.023 ± 0.007	**0.002**	0.023 ± 0.007	**0.002**
RSES	-0.023 ± 0.007	**0.001**	-0.019 ± 0.008	**0.014**	-0.019 ± 0.008	**0.019**
Log TNF-α						
CESD-R	0.002 ± 0.001	**0.055**	0.003 ± 0.001	**0.050**	0.003 ± 0.001	**0.015**
Log Leptin						
BMI (kg/m^2^)^4^	0.023 ± 0.006	**<0.001**	0.017 ± 0.005	**<0.001**	0.013 ± 0.005	**0.008**
Body fat (%)^4^	0.036 ± 0.004	**<0.001**	0.028 ± 0.005	**<0.001**	0.025 ± 0.006	**<0.001**
Log adiponectin						
SGPT (iu/l)	-0.014 ± 0.003	**<0.001**	-0.012 ± 0.003	**0.001**	-0.010 ± 0.004	**0.013**
Log oxLDL						
ΤC (mg/dl)	0.002 ± 0.000	**<0.001**	0.003 ± 0.000	**<0.001**	0.003 ± 0.000	**<0.001**
LDL (mg/dl)	0.002 ± 0.000	**<0.001**	0.003 ± 0.000	**<0.001**	0.003 ± 0.000	**<0.001**
Log oxLDL/HDL						
Insulin (μIU/mL)	0.007 ± 0.003	**0.007**	0.006 ± 0.003	**0.015**	0.006 ± 0.003	**0.040**
ΤC (mg/dl)	0.002 ± 0.001	0.001	0.002 ± 0.000	**<0.001**	0.002 ± 0.001	**<0.001**
TG (mg/dl)	0.001 ± 0.000	**<0.001**	0.001 ± 0.000	**<0.001**	0.001 ± 0.000	**<0.001**
HDL (mg/dl)	-0.378 ± 0.059	**<0.001**	-0.362 ± 0.068	**<0.001**	-0.358 ± 0.075	**<0.001**
LDL (mg/dl)	0.078 ± 0.021	**<0.001**	0.101 ± 0.020	**<0.001**	0.104 ± 0.021	**<0.001**
Log CRP						
BMI (kg/m^2^)^4^	0.030 ± 0.006	**<0.001**	0.026 ± 0.006	**<0.001**	0.025 ± 0.006	**0.008**
WC (cm)^4^	0.007 ± 0.003	**<0.001**	0.011 ± 0.003	**<0.001**	0.011 ± 0.003	**<0.001**
Body fat (%)^4^	0.022 ± 0.005	**0.012**	0.025 ± 0.007	**<0.001**	0.021 ± 0.008	**<0.001**
ALP (U/L)	0.007 ± 0.002	**0.003**	0.005 ± 0.002	**0.013**	0.005 ± 0.002	**0.036**

IL-6, interleukin-6; BMI, body mass index; VFL, visceral fat level; RSES, Rosenberg Self-Esteem scale; Revised; TNF-α, tumor necrosis factor-α; CESD-R, Center for Epidemiologic Studies Depression Scale; SGPT, serum glutamic-pyruvic transaminase; oxLDL, oxidized low-density lipoprotein; TC, total cholesterol; LDL, low-density lipoprotein; HDL, high density lipoprotein; TG, triglycerides; CRP, C-reactive protein; WC, waist circumference; ALP, alkaline phosphatase;

^1^unadjusted model, ^2^adjusted for age, gender and WC, ^3^adjusted for age, gender, WC, physical activity level, smoking and the presence of any of the metabolic disorders (hypertension, hyperglycemia, hyperlipidemia). ^4^In this analysis, WC was not included as covariate in any model. Values resulted from linear regression models. Level of significance was set as 0.05. Significant p are in bold.

Given the importance of gender dimension in obesity management, we investigated whether the above significant associations between mental/emotional health and inflammatory biomarkers exist when dividing our sample according to gender. The association that remained statistically significant was the positive one between TNF-α and CESD-R in males (Beta3 ± SD= 0.005 ± 0.002, p=0.021).

## 4 Discussion

Obesity is a multifactorial disease with a great inflammatory and OS stress burden that leads to a decreased quality of life and various physical and psychological consequences. Furthermore, it increases the likelihood of occurrence of MS as it contributes to its risk factors such as hyperlipidemia, insulin resistance, and high blood pressure. The relationship between obesity-related inflammation and OS with physical, mental and emotional health is documented, but not thoroughly investigated. Therefore, our aim was to explore the association between various inflammatory and OS markers with several disease related parameters, with a focus on quality of life, for the first time in Greek metabolically unhealthy people with obesity.

This is the first study showing an inverse association between IL-6 and self-esteem, as assessed by RSES. In past, IL-6 was associated with self-esteem (22), however this association in subjects with obesity and metabolic abnormalities hereby is novel. Self-esteem, inversely linked with obesity and MS (10), affects social adaptation and success, and it has been demonstrated that a person with low self-esteem exhibits distinct reactions to negative inputs compared with a person with high self-esteem (23). Low self-esteem leads to a social inability to develop relationships and is critical to a person’s well-being since mental well-being, social alteration, and overall quality of life may be affected (24).

Herein, TNF-α was associated with CESD-R, a questionnaire for evaluating depression. Depression is a disorder also linked to obesity in a vicious circle. The co-occurrence of obesity and mental illnesses is a logical state given their overlapping drivers, but not thoroughly elucidated mechanistically. Depression can be induced by obesity not only through psychological and environmental factors but also through peripheral low-grade inflammation, rendering the illumination of the inflammatory profile and the associated symptoms imperative (25). As mental disorders are associated with low-grade inflammation, the use of anti-inflammatory agents could be helpful when administered in patients with mental disorders and evidence of baseline inflammation (26). Higher CESD and other depression scores have been linked with a higher risk for MS (27). Interestingly, Mata and his colleagues (27) who showed that diet and physical activity may in part explain the relationship between CESD and MS, proposed that other factors (such as inflammatory mediators) may be also involved in this interplay. In 493 patients that had undergone laparoscopic gastric banding surgery, CRP was positively correlated with Beck Depression Inventory (BDI), a score that assesses the risk for depression (28). In experimental animals a link between inflammation, metabolism, and depression has been indicated, and in humans it has been demonstrated that inflammation in depression is exacerbated by obesity (29). However, studies that examine the association of inflammatory markers with psychological health and quality of life in MS subjects are scarce. Only Kim et al. (15) showed that CRP was negatively associated with the overall quality of life (as assessed by the EuroQol 5-dimension instrument) and was positively associated with mobility issues and suicidal ideation in MS. Chronic activation of the amygdala in the brain, which is primarily associated with emotional processes and depressive disorders, has been reported to induce inflammation, insulin sensitivity and eventually CVD (30). Hence, management of obesity and related metabolic abnormalities may not be the only treatment approach in presence of depressive disorders, and the treatment of the disorder itself should also be considered in terms of reducing the risk for CVD. Given that no other studies have examined the associations of other inflammatory markers with quality of life in metabolically unhealthy people with obesity, our findings regarding IL-6 and TNF-α can be considered of significant importance.

In our study when dividing the sample according to gender the positive association between TNF-α and CESD-R remained significant in male adults. Generally, it is known that sex affects the occurrence of mental health disorders possibly due to the differences in sex hormones, gut microbes and genetics. The female gender has been proposed to be more prone to developing inflammation-related mental disorders (31). Yet, a recent systematic review made the case that the mechanisms of action behind the relationship obesity/depression/female sex are not fully explored and more studies are needed (32). Our result should be interpreted with caution due to the small sample size (50 males in the analysis). Nevertheless, it is of interest as it highlights the complexity of such relationships and the various confounding factors which must be considered.

Additionally, we show herein the profile of inflammatory and OS markers in a Greek cohort with obesity and more importantly we explore the relationship of the above markers with a large number of anthropometric, biochemical and lifestyle parameters, focusing in their quality of life firstly ever. Our population presented an adipokine and OS markers profile similar to other studies in metabolically unhealthy people with obesity. CRP, IL-6 and adiponectin levels were similar to other surveys in Greek metabolically unhealthy cohorts with obesity (33, 34). Alike, levels of MPO, oxLDL and its ratios were close to the ones in non-Greek MS patients (35, 36).

Biomarkers were evaluated according to the presence or not of any metabolic abnormality (hyperlipidemia, hyperglycemia and hypertension) or according to the number of MS components that each individual exhibited along with central obesity. Interestingly, we observed a significant difference only in TNF-α and the ratios of oxLDL with lipoproteins, with higher levels in hypertension and hyperglycemia, and an increase along with the number of MS components. Hypertension and diabetes are associated with low grade inflammation and OS, supporting our findings. TNF-α is well recognized for its role in obesity and its comorbidities. It is implicated in diabetes through carbohydrate dysregulation and inhibition of insulin action, and in hypertension by reducing vasodilation and promoting sympathetic over activity (37). Particularly in hypertension, TNF-a is upregulated and the use of its inhibitors has shown promising results in both experimental and clinical studies (38). Recently, serum TNF-a was shown elevated in T2DM patients vs controls and in obese vs non-obese diabetic patients, being associated with glycemic control and insulin resistance (39). Similarly, OS markers, such as oxLDL and its ratios are usually dysregulated in both diabetes and hypertension with oxLDL/LDL being positively associated with severity of coronary atherosclerosis in T2DM patients (40). In accordance with our findings, Girona and his colleagues (35) found higher levels of both ratios in patients with MS vs control, and their significant correlation with components of MS (P < 0.001).

The relationship between different circulating biomarkers and parameters that are linked to obesity and MS was also explored. Several significant correlations occurred, with some of them remaining significant after adjusting for covariates in the regression analysis. The associations that remained significant in the final model which included adjustment for age, gender, WC, physical activity level, smoking and the presence of any metabolic disorder revealed some very interesting results. More specifically, IL-6 was positively associated with BMI and VFL and negatively with RSES. TNF-α associated with CESD-R, leptin with BMI and % body fat and adiponectin was inversely associated with SGPT. OxLDL was positively associated with TC and LDL, oxLDL/HDL with insulin, TC, TG, LDL and negatively with HDL. Finally, CRP was positively associated with BMI, WC, % body fat and ALP.

The link of circulating IL-6, CRP and leptin with obesity and metabolic abnormalities is well documented, with studies showing their positive association with different anthropometric parameters (41–43). Such link between IL-6 and VFL was expected in our study, since IL-6 is released to the circulation from visceral adipose tissue rather from subcutaneous, contributing to inflammation and insulin resistance (44). This does not stand for circulation leptin whose concentration is directly related to adipose tissue size and its secretion is higher from subcutaneous than from visceral adipose tissue (45), explaining the reported association with % body fat but not with VFL.

Adiponectin was negatively associated with SGPT and CRP positively with ALP. The literature supports that high levels of SGPT increase the risk of MS, even in healthy individuals with normal liver enzymes range and without any liver disease (46). Also, it is suggested that high liver enzymes affect liver amino acid metabolism, resulting in high levels of hepatic transamination of amino acids even before the deposition of liver fat, contributing to the pathogenesis of MS and insulin resistance (47). Adiponectin is known for its insulin-sensitizing and anti-inflammatory role which may explain the above relationship. Finally, oxLDL and oxLDL/HDL were associated with insulin and lipids as has been previously reported. In the Diné Network for Environmental Health (DiNEH) Project, which was conducted in 252 participants from 20 Navajo communities, oxLDL was positively associated with TC and LDL and oxLDL/HDL with TG and LDL, but interestingly negatively with TC (48). OxLDL/HDL correlated with insulin and HOMA-IR in 214 men with obesity (49) with the authors suggesting that these findings confirm the active role of HDL in the reverse transport of lipid peroxidation products away from peripheral tissues which is the opposite to the role of LDL (50).

The main strength of this study is that it assessed a complete profile of the participants, including validated questionnaires for the evaluation of the quality of life, as well as well-recognized methods for assessing anthropometric, biochemical parameters and circulating biomarkers. Another strength is the use of strong potential confounders in the regression analyses which allowed us to extrapolate more confident conclusions. Yet, we are aware of the limitation as regards the sample size and the cross-sectional design, which does not allow for inferring causality.

## 5 Conclusion

In conclusion, our study introduced for the first time a negative association of IL-6 with self-esteem and a positive one of TNF-α with depression in a Greek cohort with obesity. A larger sample will allow researchers to verify the interplay between inflammation and mental health in obesity-related pathologies. The profile of adipokines and OS markers was similar to other studies on obesity and metabolic abnormalities. Adipokines were associated with anthropometric measurements and biochemical parameters as expected. The understanding of the mechanism through which inflammation links obesity and its comorbidities with mental health may be very useful for the management of both metabolic and mental disorders.

## Data availability statement

The raw data supporting the conclusions of this article will be made available by the authors, without undue reservation.

## Ethics statement

The studies involving human participants were reviewed and approved by Harokopio University Ethics Committee (ID protocol: 1799/13-06-2019). The patients/participants provided their written informed consent to participate in this study.

## Author contributions

AK conceptualized and designed the study. CA, EV, S-AK, AG, KK, MA, and IS were involved in the investigation. CA performed the analysis and drafted the initial version of the manuscript. All authors contributed to the article and approved the submitted version.
